# Hôpital St. Louis

**Published:** 1829-10-01

**Authors:** 


### HOPITAL ST. LOUIS.

### DISEASES OF THE SKIN.*

Perhaps there is no class of diseases with which practitioners generally are so little acquainted as the complex and numerous affections of the skin, and, considering the limited sphere of observation presented to most medical men in private, or indeed in hospital practice in this country, the circumstance is less to be wondered at than regretted. All who have paid attention to the French hospitals, have spoken in the highest terms of the opportunities afforded at the St. Louis for the study of these diseases, and we are happy to observe that the stores of information accumulated in its walls, are likely to be made available to the profession by the publication of cases and the clinical lectures of its professors. It seems that MM. Biett and Alibert have each commenced a "cours de clinique" the most interesting and valuable parts of which will be given from time to time by M. Cazenave, in the Journal Hebdomadaire. We think we shall be doing some service to the state by dedicating a space in our hospital department to these reports. It has always been our aim, and we trust we have, in some degree, succeeded, to render this Journal a depository of practical facts, a mirror, as it were, of the "very age and body of the time," as far as medicine is concerned, and a work of something more than that ephemeral stamp which is flung aside and forgotten after the careless perusal of the day.

The first affection to which we shall allude is ACNE in its different forms.

*Case* 1. A miller, 35 years of age, of robust and sanguineous habit, who had worked hard, but never used excesses in diet, was affected five years ago with *acne rosacea* on the face, which, after repeated attacks of inflammation, ended in prominent hard tubercles, presenting all the characters of *acne indurata*. The tubercles were surrounded by much swelling and injection of the capillary system of the face, but digestion was good, and the general health unaffected. Aperient medicines were tried for some time without effect, and M. Biett determined to employ, at the same time, on opposite sides of the face, blisters and frictions with the ioduret of sulphur, the former recommended by Ambrose Paré, the latter long used with advantage by himself. Both were successful, but the blistering answered quickest and best, and the cure was complete at the end of three months and a half. The patient then returned to his employment, and in the course of five months the eruption reappeared, though not in so severe a form. There were now merely pustules without much alteration of the skin,

* Journ. Hebdom. Nos. 34—38.

and M. Biett expected that mineral acids and aperients would be quite sufficient to check their progress. In this, however, he was deceived, and blisters were again resorted to with such favourable effects, that it is hoped they will a second time effect a cure.

*Case* 2. This was an example of *acne rosacea,* following intemperance, especially in drink. Revulsives having failed, the ointment of the hydriodate of potass, accompanied with occasional local bleedings, produced the re-absorption of the pustules, diminution of the capillary injection, and, finally, a return of the healthy colour in the face.

In his clinical remarks on these cases, M. Biett admitted the four-fold division of Willan into acne simplex, acne punctata, acne indurata, and acne rosacea. Though certainly distinct, yet they frequently run into one another, especially the three first, and M. Biett thinks that it is useless or worse to consider them as separate diseases, as all are essentially seated in the small follicular glands. With respect to the treatment, M. Biett calls in question the efficacy of purgatives and tonics so usually recommended, and believes that we should have recourse to topical means. In some very severe cases the disease has entirely yielded to blisters; and acid lotions, as that with the hydrochloric acid, lotions of bitter almonds, &c. are frequently of service. M. Biett has obtained very good results from the employment of resolvent ointments, especially that of the ioduret of sulphur, but mercurial washes, the red wash, Gowland's lotion, he has never seen of service. Cauterization with the nitrates of mercury or silver leave deep cicatrices behind them, and are frequently attended with serious accidents. M. Biett proposes to return hereafter to the particular variety of acne rosacea.

### Lupus.

Three cases of this disgusting looking malady are detailed, and each present some peculiar points of interest. In the first the disease was exclusively local and unattended with any general symptoms of scrofula. The inferior part of the face was affected, and the eruption was singularly modified by an attack of small-pox, which the patient had whilst in the house. The cicatrix, however, subsequently gave way, and the disease has re-appeared. M. Biett remarks, that the condition of the cicatrix in lupus will discover to a person accustomed to such cases whether or not the disease will return. If it is unequal and traversed by bands we should fear such an occurrence; but if smooth and soft, it will not give way. We suppose that this observation is to be taken with some degree of limitation, because, be the cicatrix as perfect as it may, it must still be more prone to suffer afresh from the disease, than the original sound skin.

The second case is alluded to as shewing the inefficacy of the preparations of iodine, although so much lauded in scrofula of late. The patient had taken the tincture for 142 days, without the least amendment. We wonder he could take it so long at all.

The third case was that of a young man affected with scrofulous lupus, who was once cured by the operation for making a new nose, but in whom the disease had returned. It originally began three years ago in the form of a tubercle of the mucous membrane of the nostrils, which extended from within outwards, and invaded the alæ nasi, a rare instance of lupus commencing in the mucous texture. The right cheek became discoloured, tubercles formed in it, ulcerated, and the malady spread, when the patient was received in the hospital of Delpech, who cauterized the cheek with the nitrate of mercury, and the nose with the nitrate of silver. The improvement being temporary only, M. Delpech performed the operation for a new nose with such success, that in twenty days cicatrization was complete. However, as has been mentioned, the patient is now in the St. Louis with a return of the complaint, which occurred after the expiration of two years in the right cheek, then attacked the left, and subsequently invaded the internal surface of the artificial nose, from which it has spread to the external. The nose itself is round, prominent, not much deformed, and M. Biett is treating the complaint by cauterization.

PORRIGO.

A young man, about three years ago, having very little hair, bought a second-hand wig, and shortly afterwards the arms and legs became affected with an eruption, having all the characters of porrigo favosa. Subsequently the hairy scalp became affected, and on admission into hospital, yellow favous crusts depressed in the centre, existed on these parts. By means of frictions with the ioduret of sulphur, the disease disappeared, and at present no more than discolouration and injection of the skin remain. The case is reported as one of contagious origin, and the unfortunate wig is made both by patient and doctor to bear all the onus and shame of the business. The first appearance of the eruption on the arms and legs might seem to militate against this view, seeing that wigs are most commonly worn upon the head. The patient, however, helps out this lame part of the *contagious* theory, for he states that he often lost the wig in his sleep, and found it on awaking sometimes on one side of him, sometimes on another; and besides, says the reporter, the disease might have existed first upon the head, without its attracting the patient's attention. Seriously, we think that the idea of contagion, in this instance, is founded on very limping kind of evidence, if no better could be got than that which has been published.

In another case, which had just been dismissed, the porrigo was extremely diffused. Not only the scalp, but the trunk and limbs were covered with large favous crusts, and yet the patient preserved all his physical powers. The treatment consisted in the application of alkaline remedies externally, which effected a perfect cure, the scabs dropping off, and nothing remaining but discolouration of the parts beneath.

PSORIASIS.

A seaman, ætatis 24, after three paroxysms of intermittent fever, was suddenly attacked with an "excentric explosion" on the skin, which was almost immediately succeeded by psoriasis diffusa. The scales became detached, and a vesicular eruption was established on the patches themselves, which gave the whole disease an appearance quite different from the scaly affections. Under the influence of baths and light mineral acids, the vesicular eruption disappeared, and the psoriasis returned. M. Biett observed, that he has frequently seen these squamous diseases of the skin succeed intermittent fevers, especially when connected with lesion of the hypogastric viscera; lepra and pityriasis have frequently shewn themselves under these circumstances. In another patient in hospital the disease began as psoriasis gyrata, and ended in psoriasis diffusa. In a third patient affected with simple psoriasis, M. Biett employed the "dento-chloruret of mercury," (the corrosive sublimate we suppose) in doses of the one-twentieth of a grain, up to the amount of thirty grains. The disease, which had existed for upwards of a year and a half, completely yielded to the medicine, but has since returned.

SCABIES.

In a patient who had laboured under itch for five months, M. Biett made trial of frictions with oil, in the manner recommended by M. Delpech. The itching ceased in a very few days, and the vesicles gradually subsided, a result which has followed this treatment in several instances. M. Biett intends to institute experiments on a scale sufficiently extensive to determine the value of the treatment. We fear that nothing will answer like sulphur, but the experiment is worth trying.

M. Biett disagrees with Bateman in regard to that not very unfrequent affection *sycosis menti*. Bateman has withdrawn it from the order of pustules and placed it under that of tubercles, in which M. Biett thinks that he is wrong. The disease, according to the latter, begins as psydracious, acuminated, distinct pustules succeeding one another, and only ending in tubercles after repeated attacks of inflammation.

### XXXIII.

### HOPITAL ST. LOUIS.

#### 1. SYCOSIS MENTI.*

Pursuant to our intention already expressed in this Journal, we resume the notice of the reports from the St. Louis Hospital, by M. Cazenave. Every man who feels an interest in his profession, must regret that no similar institution for diseases of the skin exists in this metropolis. At present these cases, and numerous certainly they are, are scattered throughout so many dispensaries ard hospitals, that scarcely any practitioner can hope to become sufficiently acquainted with them. No doubt, that if a medical man will make it his business to hunt them out, he will find them in abundance, even under existing regulations and impediments, a fact which the admirable works of Willan and Bateman are sufficient to establish. But the consumption of time in such a proceeding is so enormous, and the advantages, in a certain point of view, so problematical, that we entertain no hope of our confrères, young or old, engaging in the undertaking, at least until this good city of London becomes the Utopia of which dreamers love to talk. All we can do then is to make the most of what we have at home and what we can import from abroad!

There being many cases of *sycosis menti* in the Hospital, they have been made the subject of a Clinical Lecture by M. Biett. Passing over his objections to the term *sycosis* (the affection does not always resemble a *fig*,) as well as to that of *mentagre*, substituted for it, we believe, by Alibert, we may stop for a moment to consider the ravages it seems to have committed on the chins of the Roman dandies, when it first appeared in Italy, about the middle of the reign of the Emperor Claudius. Pliny describes it as always extremely acute in its progress, attacking all the face, the back, and the shoulders; altering the features; and giving such a hideous aspect to the patient, that death itself was considered preferable. Pliny believes that it was brought into his country by the Romans, who travelled into Asia, the greater part of which was then under their dominion. As M. Biett, however, observes, it is much more likely that the adoption of Asiatic effeminacy and luxury were the causes of the disease, than its direct importation. We all know that after the days of Lucullus, the victorious and voluptuous General of the legions of the East, the simple habits of the Romans quickly passed away; and *sycosis* was only one of the many scourges, offspring of their vices and debaucheries, that at length made the mistress of the world a prey to horde after horde of barbarians, and ended in her glories becoming no more than "a school boy's tale."

The authors of the 15th and 16th century confounded sycosis menti with other eruptions, lichen and impetigo. Willan, Bateman, Macartney, and Plumbe are wrong according to M. Biett in placing it amongst the tubercular diseases. "It is evidently a pustular eruption, for in the first place the tubercle is not constant; and in the next, in order that it may exist, the base of the pustule must have been the seat of successive inflammations, which not only indurate the dermoid tissue, but the lamellar beneath it. Tubercle then is not the essential character, and sycosis menti is a pustular affection." *

The eruption is preceded by local tension, small red points supervene, and on the 3d, or 4th day, a small and

---

* *Journ. Hebdomadaire, No.* XLII.

slightly acuminated pustule is observed. Sometimes this extends and grows more prominent, sometimes it is formed around the hair, and the latter pierces its centre. After four or five days the pustules break, slight crusts succeed, and these become mixed with superficial scales which cover the neighbouring parts. If the patient is young and healthy, the pustules are numerous, and resolution is speedy and complete. If old, or flabby, they are few in number, gradual in their progress, and imperfectly resolved. Elevations remain where the pustules were, and if fresh inflammation comes on, the lamellar tissue is attacked, and tubercle is developed. In general, sycosis menti has a regular progress, eruptions of twelve or fifteen pustules, or more, appearing every five or six days. M. Biett believes that the disease has its seat in the follicular glandules, differing, however, from *acné* in this, that whereas the latter selects parts free from hair, sycosis attacks those which abound in it.

This disease is most commonly met with in men, but occasionally it shows itself in females, and then M. Biett thinks that it resembles acné. It is most frequent after the age of puberty, and chiefly affects those with strong, brown beards. It is sometimes connected with irritation of the digestive organs, as where it follows the abuse of stimulating liquors. Persons exposed to a strong fire, as cooks, metal-workers, &c, are peculiarly liable to the disease. We have seen it apparently the consequence of privations in a miserable mechanic, where probably bad food had been assisted in its operation by worse drink. In persons disposed to its occurrence, the irritation of the razor, no doubt, would aggravate, but M. Biett conceives it to be merely a delusion of the patient's amour propre, to assign it as the cause of sycosis.

From impetigo it may be distinguished by the pustules of the latter being assembled in groups, superficial and irregular. They break spontaneously on the third or fourth day, and are replaced by a thick and extended crust. Those of *sycosis* are separate and deeper, "have evidently their seat in the sebaceous follicles," and exhibit a regularly rounded form. Eczema and herpes cannot well be mistaken by any moderately informed person for this disease, but the tubercles of syphilis are more likely to be so. The main instrument of diagnosis in the latter case, must consist, we imagine, in the history and presence or absence of other symptoms of the venereal poison. M. Biett, however, points out the specific differences between the tubercles of sycosis and those of syphilis, and remarks that the latter are primitive, prominent, large, round, shining, and of coppery or livid colour. Those of sycosis, on the contrary, are always secondary to pustules, and frequently intermixed with such; they depend on a deep inflammation of the skin, and are conoid in their shape. M. Biett relates a curious instance of the consequences that might have followed a mistake in the diagnosis of this affection. It occurred in a clock maker, who suffered from sycosis to such a degree that the tubercles had acquired an enormous size, and the case was not at all understood. After a great variety of treatment, a celebrated surgeon decided that it was cancer and proposed extirpation of the lip. Before consenting to this formidable operation, the patient consulted several other medical men, and amongst them M. Biett, who recognised the disease, and was able to effect a perfect cure.

The duration of the disease is extremely uncertain, and not to be predicted from the aspect it assumes at first. Sometimes it sets in with much severity and terminates notwithstanding in a few weeks: whilst at others, there are merely five or six pustules at distant intervals, which are very intractable indeed.

The treatment must be varied according to the circumstances of the individual case. If the patient be young and full of blood, the affection acute and extensive, bleeding, both general and local, is required. The general depletion should go first, the local succeed it, and the latter should be made *beyond* the inflamed surface. We lately saw a good example of the advantages derived from the application of leeches, in the immediate neighbourhood of the sycosis. Depletion does not answer so well in persons of a flabby lymphatic habit, when the tubercles are numerous and near one another. If they become red and inflamed, local bleeding only must be had recourse to.

When the digestive organs are in a good condition, which, by the bye, we believe they seldom are, light purging by calomel, castor oil, the acetate of potass, sulphates of magnesia and soda, &c. is useful. The local treatment must be also regulated by the nature of the case. In the pustular stage emollient applications alone should be employed, especially "the feculæ of rice, potatoes, or merely lotions of eau de guimauve." Poultices of linseed meal frequently give rise to a psydracious eruption amidst the sycosis, which becomes a very troublesome affair. When the pustules have passed into tubercles, *douches* of vapour for ten minutes, or a quarter of an hour, are often very useful. If continued longer than the time mentioned, they are apt to excite erysipelatous inflammation in the part. Sulphurous douches *en arrosoir*, and alkaline douches produce the same effect. Ointments of the proto-nitrate of mercury, in the proportion of one part in twenty-four of lard; or the ammoniacal proto-chloruret; or of the deuto-ioduret of mercury; or finally, the ioduret of sulphur, are often of service. The patient's diet is the main thing after all, and must be light and unirritating in robust patients, but even slightly stimulating in the chronic stage of the complaint in weakly persons or old men.

We need scarcely say that the above is a very excellent, practical series of observations on sycosis, and one which is not unworthy the attention of our readers. The affection to be sure is seldom severe, but by being mistaken and improperly treated may be made much worse than it would otherwise become.

II. Lupus — Frictions with the Deuto-Ioduret of Mercury.

M. Biett has seen much advantage from frictions with the deuto-ioduret of mercury in lupus, the surface becoming "reanimated" under its use, and the violet tint changing for a florid and brighter colour. From ten grains to a scruple is the quantity, mixed with lard, or any fatty substance. A scrofulous child in the hospital affected with lupus in a very severe degree, has been treated by this application, in combination with the muriate of lime internally. The ointment was only applied on the right side of the face, in order to determine its actual effects. Upwards of a month has now elapsed since the commencement of the treatment, and the left side exhibits no apparent change, but the right is evidently improved. The violet colour is replaced by one of a bright rosy red, the injection of the venous capillaries is considerably diminished, and healthier action being thus set up, strong anticipations of a cure are entertained. Iodine had been previously employed in this case without effect, but in others M. Biett has seen it productive of decided benefit.

III. Erythema Circinnatum. There is a species of erythema, not described by authors, but resembling in form the *herpes circinnatus* of Willan. It is a kind of centrifugal erythema commonly occupying the face and cheeks, presenting neither papulæ nor vesicles, and consequently neither lichen nor herpes. It is a species of puffing up of the skin, with pretty intense redness, formed into completely circular patches, the centre of which is sometimes unaffected. M. Biett has given to this variety the name of erythema circinnatum, but nothing is said about its treatment nor etiology.

IV. Rubeola — Bad Effects of Bloodletting.

During the last year a "murderous" epidemic measles prevailed at Paris, being complicated with inflammation of important organs, and almost always pneumonia. According to M. Biett the great difficulty in managing such cases consists in the employment of sanguineous evacuations. Even in the cases complicated with peripneumony M. Biett doubts their good effects, as in four children whom he saw under such circumstances, they were followed by very unfortunate results. Amongst other cases, the following occurred. A lad about 13 years of age was attacked by measles, which exhibited from the commencement spots of purpura, generally a formidable symptom. On the sixth day pneumonia supervened, bleeding was had recourse to and the patient died. In the same house, a girl of 15, *who had had the disease the year before*, and was kept away from the former patient, was nevertheless attacked with measles. From the third day there was dyspnœa, constant cough, and all the symptoms of inflammation of the right lung. Bleeding was employed — the measles quickly disappeared—the skin grew pale — the pneumonia too made progress, and this patient also sank. The last fact is cited by M. Biett as especially remarkable, in consequence of its shewing the repulsion of the eruption of measles, an occurrence which is denied to take place by authors of considerable merit.

The above cases are brought forward by M. Biett, to prove the pernicious effects of bloodletting, but unfortunately he tells us of no substitute. If acute pneumonia supervenes upon measles what is the practitioner to do? If he neglects to bleed, the patient will probably die, and then what an opportunity for the *charitable* remarks of his professional brethren, and the suspicions of relatives and friends? If he bleeds, he does wrong, according to M. Biett, and thus he is placed on the horns of a dilemma. We must confess, however, that we should be greatly disposed to adopt the depleting plan, with caution and circumspection of course, in spite of the above unfortunate cases. In these we see no evidence that the patients would not equally have sunk had the lancet never been unsheathed, nor yet that the latter was the cause of death. It does not follow because a patient with pneumonia dies *after* bleeding, that he necessarily died *of* it. Other circumstances must be adduced in order to bring the danger home to the measure, and those circumstances we humbly conceive, are not detailed in the present cases.

---

### XXXIV.

### GLASGOW ROYAL INFIRMARY.

### I. DISEASE OF THE MAXILLARY SINUS.*

### HOPITAL SAINT LOUIS.*

### I. LICHEN AGRIUS.

SCARCE a day elapses without convincing us of the importance of studying the diseases of the skin, as well as of the very indifferent manner in which they are understood in general. Not a week ago we witnessed a terrible specimen of ignorance and neglect, in the person of a female child, whose face was covered with porrigo favosa to a frightful degree. The medical practitioner had christened it *scrofula*, and, under the cover of that bugbear, deceived the parents and screened himself. Really, is it not shocking that the health and the happiness of patients should be at the mercy of impudent ignorance like this, and that men should be hardy enough to go abroad and prey, without dread of molestation from the law, on the persons and pockets of unhappy, unsuspecting victims? So, however, it is, and medical men in general have been far too agreeably occupied in the perusal of ribald lies and profitable slander, to attend to the dull, dry lesson of professional information. The present report opens with the case of a patient, of delicate habit, affected with a papular eruption having all the characters of *lichen agrius*, principally situated in the lower extremities. The papulæ were approximated, confluent, ulcerated at their apex, furnished a pretty abundant exudation, and were accompanied at times with violent itching. The disease had first appeared in childhood, and had disappeared and returned several times. Shortly before the present attack it had yielded rapidly to emollients, but the patient being a public-house-keeper, and probably, like Dennis Bulgruddery, one of his own best customers, the cure was by no means of a permanent character.

M. Biett remarked, that lichenoid affections are amongst the most severe and obstinate of the eruptions, on account of the generally deep alteration of the skin, and disturbance of the internal functions. M. Biett does not consider *lichen agrius* as a very distinct species of the lichen family, since it arises from the inflammation of another form. In fact, the papulæ first inflame, their apices then become elevated, and an exudation, more or less considerable, is established, which dries and furnishes scales, which themselves fall off and alternate with a fresh exudation. Lichen agrius is always accompanied with severe local symptoms; the patients are

* Journ. Hebdomad. No. 39, 44.

tormented with a burning heat, and the itching is almost insupportable, especially in the night, or after eating certain kinds of stimulating food, or sometimes even Seltzer water. The want of sleep thus produced is frequently very distressing.

Lichen agrius attacks all ages, appears at all seasons, but is more severe during the Winter, and appears to be aggravated by artificial warmth. It attacks indifferently either sex, but is met with most frequently in persons with rough, sallow skins, and bilious temperament, although those with fair complexions are not exempt from its visitations. Like most of the eruptive diseases, it is particularly met with in the lower classes immersed in filth and misery, and addicted to unwholesome food and drink. The eruption most likely to be confounded with lichen agrius, is the *eczema chronicum*, but the former is always characterised by papulæ, either simple or ulcerated; besides which, the skin is dry and so thickened that it is impossible to pinch it up into a fold. The surface in *lichen agrius*, is so dry that it frequently shews not the least moisture in the vapour bath, when the neighbouring parts are in a state of perspiration. The slightest examination is sufficient to distinguish this disease from any of the other eruptive affections, especially from psoriasis.

So much for the characters and diagnostic marks of lichen agrius; the following are the medicinal instructions afforded by M. Biett. As there is commonly some chronic irritation of the primæ viæ, demulcent draughts, quiet, and a light regimen, should be combined with some plain warm baths. The more active baths should be avoided till resolution is taking place, when certain quantities of the subcarbonate of potass, or even the hydro-chlorates of soda and potassa may be added with advantage. If employed too early, the medicated baths, not excepting the sulphureous, only aggravate the symptoms.

### II. LICHEN GYRATUS, AND LICHEN CIRCUMSCRIPTUS.

In the first form, *lichen gyratus*, which is very rare, and first described by M. Biett, the patches of lichen are disposed in spiral forms, like riband, contorted on themselves. Nothing more is said about it. The *lichen circumscriptus* has been confounded with *herpes circinnatus*, but in lichen it is papulæ which succeed each other, and they are full and red, especially when much itching prevails. In herpes circinnatus, on the contrary, we find not papulæ, but vesicles in all its stages, and often when the circle of herpes is large the centre returns to its natural state, which is never the case in lichen. This circumscribed form is much less severe than lichen agrius, and neither have any defined duration, whilst lichen simplex seldom lasts more than two or three weeks, though apt to recur several times.

### III. HERPES.

M. Biett and M. Alibert, both men of "consular dignity," and colleagues in the Hôpital St. Louis, would seem like many of their confréres on this side of the water to be at open war. The former is an admirer of the English School, a cautious and able disciple of Willan and Bateman; the latter is, like the Irishman, a follower of no one but himself. M. Alibert accuses M. Biett of Anglo-mania—the world accuse M. Alibert of something worse, a spice of quackery! For our own parts, being islanders and fully impressed with the excellence of our own school, we reject M. Alibert and cleave to M. Biett, or rather to his doctrines. Herpes in the French, i. e. the *Alibertin* code, corresponds with *dartre*, and to the latter term M. Biett takes strong and just grounds of exception. As the word, however, is not prevalent amongst us we are under no necessity for proving it a bad one, and therefore pass on to more practical matters.

The word *herpes* is universally admitted to signify a vesicular eruption, of which there are many varieties. *Herpes phlyctænodes* is characterised by isolated clusters, separated from each other by intervals of sound skin. The first symptom is smarting, then small red spots make their appearance, which approach each other, raise the epidermis, contain a light coloured serum, and assume the vesicular character. At the expiration of five or six days the vesicles open, the liquid escapes after having acquired a slight opalescence, and nothing more than redness covered by superficial scales remains behind. This affection might be confounded with *pompholyx* when the bullæ are of small extent, and especially with pompholyx *pruriginosus*. But however small the bullæ may be in pompholyx, they are still sound, whilst in herpes phlyctænodes if the vesicles are large they are also irregular and angular. The bullæ of acute *pemphigus* differ too widely from herpes, to allow of a mistake between them being made. In *eczema* the vesicles are rarely disposed in groups as in herpes phlyctænodes, but even when they are so, as in two cases seen by M. Biett, the vesicles are much smaller, less prominent, and the redness more intense than in the latter affection. The duration of herpes phlyctænodes may exceed two or three weeks, but it varies greatly; the redness remains for several months.

*Herpes Zoster* is by no means, as has been stated, peculiar to the trunk, but may occupy the thigh, the haunch, the toes, the forehead, the hairy scalp, the neck, the cheeks, even the mucous membrane of the mouth. Its direction too may be oblique or horizontal, as it extends upwards from the middle of the trunk, or downwards to the lower extremities. The term *zoster* is not therefore strictly correct. It has reigned almost epidemically during the present year, and a great number of cases have been received in the Saint Louis. Until the present year M. Biett had almost always remarked it on the right side of the body, but during the epidemic the left side has been almost exclusively affected, a curious occurrence enough. The foregoing doctrines or observations will be considered, in many respects, heterodox in this country, but facts are stubborn things. The symptoms are nearly the same as those of herpes phlyctænodes, the vesicles becoming opalescent on the 5th or 6th day, approaching each other but not in general coalescing. In some rare cases they mix and form irregular bullæ, a circumstance which by no means justifies the placing the disease amongst the latter order. Sometimes the vesicles become purulent, break, and form genuine ulcers, which happens especially in elderly men with weakened constitutions. The ulceration may almost be predicted by the violet tint of the eruption and the translation of the vesicles into the purulent state. The ulcers continue open for two or three weeks. Herpes zoster is always recognizable, whatever region it attacks, by the disposition of its clusters; its causes are extremely obscure. Most commonly it is to be considered as a trifling affection, and the prognosis framed accordingly. The treatment consists in the employment of diluents (délayans,) gentle laxatives, and baths. When its irruption is attended with much local heat, camphorated ointment is a good application. The employment of caustics lately recommended by M. Bretonneau and others on the Continent is occasionally applicable to the very early stage of the complaint, and they (the caustics) should be mild ones.

*Herpes circinnatus*, frequently confounded with the porrigo scutulata, or *ring-worm*, is characterised by circles bordered by vesicular surfaces successively developing themselves, one beyond the other, whilst the centre is generally sound. M. Biett attended a lady last year, who was first affected with a single circle of herpes circinnatus on the thumb; in the course of a week or two the eruption had extended to the fore-arm but still preserved its circular character. The disease may last for months, and leaves behind it marks which are evident long after itself has disappeared. The circular form and vesicular nature of herpes circinnatus are sufficient to distinguish it from lichen circumscriptus, the only affection with which it is liable to be confounded. The treatment consists in the exhibition of acidulated drinks, simple and vapour baths. If the disease is rather severe, local bleeding by leeches, &c. beyond the inflamed circle, or even in the centre when extensive, may be practised with advantage.

*Herpes iris*, characterised by the presence of two or even three vesicular circles inclosed within each other, is so rare that M. Biett has only witnessed two cases, notwithstanding his extensive field for observation.

*Herpes labialis*, as every body knows, is commonly critical of a catarrh, or a fever, or indeed any illness, and is of no sort of consequence, except to young ladies and very particular gentlemen. It quickly disappears.

*Herpes præputialis* is distinguished by vesicular groups most frequently situated on the external skin of the prepuce, sometimes on its internal surface, or even on the glans. It is attended by a considerable secretion from the follicular glandules of an unctuous fluid. The vesicles break, are detached in small rolled membranes, and leave behind the cutis vera denuded and ulcerated. M. Biett remarks, as has indeed been frequently remarked before, that herpes præputialis is constantly mistaken by ignorant practitioners for syphilitic sores, with the most unfortunate consequences, of course, to the patient. Attention to the appearances and history will enable a well informed man to distinguish them without much difficulty. Herpes præputialis often assumes a chronic character, especially when stimulating or caustic applications have been used; it then becomes extremely intractable, lasting for months, or even several years, and is accompanied with phymosis. The abuse of venereal pleasures is a frequent cause of the disease, and so is the leucorrhœal discharge. One of the greatest difficulties encountered in the treatment of chronic herpes is the concealment of the glans, to obviate which M. Biett dilates the præputial orifice by means of sponge tent. Surely, however, the operation for phymosis must be preferable to so painful and tedious a measure as dilatation, at least if the case is at all severe. The remainder of the treatment consists in the employment of soothing applications, alkaline baths, and sometimes gentle stimulating lotions of the sulphate of alum, zinc, or copper.

### IV. Eczema.

Many specimens of this vesicular disease being found in the hospital, have furnished *fulcra* on which M. Biett has rested some clinical remarks. In the beds, No. 6, and 17, lay two patients affected with chronic eczema. The first was a cutler at the decline of life, and of feeble constitution, in which the disease had been severe from its commencement, had resisted every measure had recourse to, or if checked for a time had always re-appeared. M. Biett remarked that eczema is always most severe in old people, and in weakly constitutions, since evacuations of blood are productive of mischief in such cases. The present patient is treated by preparations of sulphur internally and externally also in the form of baths. The second case was that of a shoemaker, in whom the eczema commenced four years previously with extreme severity, but yielded notwithstanding to a very stimulating treatment, namely, bitters, "*vin antiscorbutique*," &c. The return of the disease was clearly referrible to the salt provisions on which the patient lived.

In another case eczema occupied the hairy scalp, a situation in which it is very obstinate, and not unfrequently confounded with porrigo. By recollecting the vesicular nature of eczema we can always distinguish the diseases. Even when the primary state of the eruption is obscured by scales, the latter will readily fall off under the application of poultices, and the vesicles be reproduced. This patient is taking arsenical preparations with much advantage.

In a fourth case, a crop of papulæ appeared upon the surface occupied with the vesicles of eczema, a circumstance that might be mistaken for the conversion of the papulæ into the vesicular disease or *vice versâ*. Another example of a complication of eruptions was presented in the person of a man in whom *eczema rubrum* was combined with roseola, both of which pursued their regular course. The exanthema occupied almost the whole of the integument, whilst the eczema was confined to the nipple, a spot in which it is extremely obstinate. Such coincidences as these are believed by M. Biett to have deceived Willan, who mentions *lichen agrius* as passing into *impetigo*, &c.

V. PSORIASIS.

In a case of psoriasis diffusa, where arsenical preparations failed in toto, those of sulphur were attended with remarkably good effects. M. Biett considers this as a curious fact, since they commonly are not so fortunate, especially in chronic cases which have not succumbed to energetic treatment.

VI. ELEPHANTIASIS ARABUM.

In the bed, No. 11, was a carrier, 70 years of age, who had suffered from this disease in the left leg and foot, for forty years. These parts were double their natural volume, hard, and much deformed; the tumefaction ceased abruptly at the junction of the middle with the upper third of the leg. The disease had commenced with repeated attacks of erysipelatous inflammation, which succeeded each other without interruption for several years, and ended in deep induration of the cellular tissue, and a scaly, indeed almost horny thickening of the epidermis. This disease, the elephantiasis *Arabum*, generally attacks the lower extremities, though in India it has been seen to occupy the scrotum. It begins by erysipelatous inflammations, which succeed each other at longer or shorter intervals, and the lymphatic vessels are ordinarily implicated, and form a kind of cord extending up to the glands of the groin. The erysipelatous attacks are accompanied by symptoms of general fever, symptomatic, in M. Biett's opinion, of the local disease. The inflammatory action attacks the cellular tissue and lymph is thrown out, especially on the dorsal surface of the foot, producing enormous increase of volume and burying the nails, which almost disappear. Sometimes tubercles supervene, at others a vesicular eruption, or a considerable and horny thickening of the integuments.

If the disease is recent we may hope to subdue it; if not, the prospect is a very bad one. The therapeutic measures which M. Biett has most frequently found to succeed, have been bloodletting, if the patient is robust—light revulsives — rather stimulating *douches*, either sulphurous, alkaline, or vapour—but more than all, compression, from which he has obtained unexpected success.

We must here conclude this report, which we have been at some pains to condense from the originals. If it serve to convey information on the diseases of the skin to any of our readers, we shall deem our labour amply repaid.

